# Clinico‐sero‐pathological characteristics of anti‐Ha antisynthetase syndrome

**DOI:** 10.1111/bpa.13319

**Published:** 2024-11-18

**Authors:** Bing Zhao, Ying Hou, Kai Shao, XiaoTian Ma, YaPing Yan, Jian‐Qiang Lu, Wei Li, ChuanZhu Yan, LiNing Zhang, TingJun Dai

**Affiliations:** ^1^ Department of Neurology Qilu Hospital (Qingdao), Cheeloo College of Medicine, Shandong University Qingdao Shandong China; ^2^ Department of Neurology, Shandong Key Laboratory of Mitochondrial Medicine and Rare Diseases, Research Institute of Neuromuscular and Neurodegenerative Disease, Qilu Hospital of Shandong University Jinan Shandong China; ^3^ Department of Medicine Experimental Center Qilu Hospital (Qingdao), Cheeloo College of Medicine, Shandong University Qingdao Shandong China; ^4^ Key Laboratory of the Ministry of Education for Medicinal Resources and Natural Pharmaceutical Chemistry College of Life Sciences, Shanxi Normal University Xi'an China; ^5^ Department of Pathology and Molecular Medicine Division of Neuropathology, McMaster University Hamilton Ontario Canada; ^6^ Department of Rheumatology Shandong Key Laboratory of Medicine and Prevention Integration in Rheumatism and Immunity Disease, Qilu Hospital of Shandong University Jinan Shandong China

**Keywords:** anti‐Ha antibody, favorable outcomes, myositis‐specific antibodies, necrotizing myopathy without perifascicular pattern, relapse

## Abstract

To define the clinical, serological, and muscle histopathological characteristics, as well as treatment outcomes, of patients with anti‐Ha antibody. We performed a retrospective analysis of clinical, serological, and pathological data and long‐term treatment outcomes of anti‐Ha patients between January 2005 and July 2023 at our center. Anti‐Ha antibody was identified by immunoblot and reconfirmed by immunoprecipitation. Of the 570 patients with idiopathic inflammatory myopathies, 17 (3.0%) were found to be anti‐Ha positive, of whom 5 (29.4%) were also positive for another myositis‐specific antibody (MSA). All patients with anti‐Ha antibody as the single MSA (12/17, 70.6%) had clinical and histopathological evidence of muscle damage. Skin lesions were identified in nine of them (75%), while both interstitial lung disease and Raynaud's phenomenon were only seen in four patients. A necrotizing myopathy without a perifascicular pattern was the most common pathological manifestation (50%). Perifascicular necrosis (PFN) and myofiber major histocompatibility complex class‐II expression were observed only in one and four patients, respectively. Muscle weakness relapse was reported in five patients, and skin rashes worsening were observed in one patient. Most of the anti‐Ha patients (66.7%) finally achieved a favorable outcome at last follow‐up. Anti‐Ha antibody might not be as rare as previously thought and may coexist with other MSAs. Muscle damage is the most common manifestation in anti‐Ha patients, while extra‐muscular symptoms except for the cutaneous manifestations are unusual. The histopathological features varied with a predominance of necrotizing myopathy without PFN. These patients often finally had favorable outcomes, although relapses often occur.

## Graphical abstract

A necrotizing myopathy without a perifascicular pattern is the most common pathological manifestation of anti‐Ha ASS.

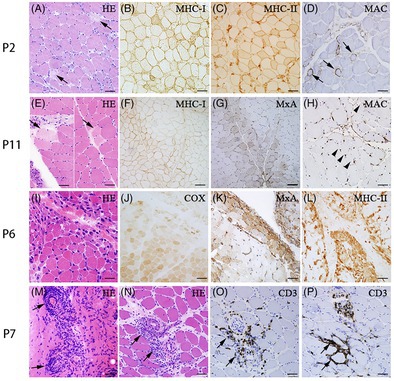

## 1 INTRODUCTION

Idiopathic inflammatory myopathies (IIMs) are a heterogeneous group of autoimmune disorders, characterized not only by the skeletal muscle damage but also by a wide variety of extra‐muscular manifestations [1, 2]. Anti‐synthetase syndrome (ASS) is now recognized as a distinct subgroup of IIMs defined by the presence of the autoantibodies against the cytoplasmic aminoacyl tRNA synthetase (anti‐ARS) plus at least one of the clinical features, including interstitial lung disease (ILD), myositis, arthritis, Raynaud's phenomenon (RP), fever, mechanic hands, and skin lesions [3, 4]. And the high frequency of extra‐muscular symptoms was seen as a prominent clinical feature of patients with ASS [5].

Moreover, myositis is proposed as one of the classic triad in ASS [5, 6], and the muscle pathology of this IIM subgroup has been increasingly investigated in recent years. Perifascicular necrosis (PFN) is considered a distinct characteristic of ASS, especially in anti‐Jo‐1, anti‐PL‐7, and anti‐OJ patients [7, 8], but this pathological feature is also common in anti‐Mi‐2 dermatomyositis (DM) patients [9]. The presence of human leukocyte antigen (HLA)‐DR with a perifascicular pattern without MxA expression is also recognized as a distinct pathological biomarker in ASS, most commonly in anti‐Jo‐1 myositis [10], while this is also described in other subtypes of IIMs [11].

Since the two novel anti‐ARS antibodies were recently reported in 2022, there have been 10 anti‐ARS autoantibodies identified up to now [12]. Each anti‐ARS antibody seemed to represent a different clinic‐pathological phenotype [4, 6, 13, 14, 15]. Specifically, ILD and myocardial injury were the prominent clinical features of anti‐OJ patients [6]. A moderate muscle damage with less capillary dropout on muscle biopsy was identified in anti‐PL‐7 individuals [16], while a cluster of amyopathic DM, Sjögren's syndrome with chronic‐type ILD seemed to be the clinical prototype of anti‐KS subgroup [15]. Anti‐Ha (tyrosyl‐tRNA synthetase) antibody was identified firstly in 2005 in a patient who had several features consistent with ASS [17]. However, only a few cases with anti‐Ha antibody have been reported from then on [18].

In the present study, we demonstrated the clinical, serological, and muscle histopathological characteristics, as well as the long‐term treatment outcomes, of 12 patients with anti‐Ha antibody as the single myositis‐specific antibody (MSA). Anti‐Ha antibodies were identified by immunoblot combined with a blocking test and reconfirmed by the immunoprecipitation (IP)‐western blotting (WB) procedure. Moreover, we compared the clinico‐pathological differences between anti‐Ha and other anti‐ARS subgroups.

## 2 PATIENTS AND METHODS

### 2.1 Patients

This is a retrospective observational single‐center study. There were a total of 906 patients diagnosed as IIMs according to the criteria proposed by the European Neuro‐muscular Centre (ENMC) at our neuromuscular disease (NMD) Center from January 2005 to July 2023 [19]. After exclusion of the individuals with sporadic inclusion of body myositis (16 patients) and those who did not have sera samples available for detection of MSAs and myositis‐associated antibodies (MAAs) (320 patients), 570 patients were finally included in this study. Among these patients, individuals who tested positive for any one of the anti‐ARS antibodies and did not possess any other MSA were classified as having ASS and subsequently included in this study [20].

In regard to clinical assessment, muscle strength was evaluated by the ordinal 10‐point (0–10) manual muscle testing (MMT) scale [21]; asymmetric muscle weakness was defined as a difference of 2 or more MMT grades between the two sides of the same muscle group. Extra‐muscular global activity was measured by the physician based on a 10‐cm visual‐analogue scale (VAS) [22]. Treatment response on muscle strength was graded as follows: no improvement, mild improvement (up to 2 grades improvement in one to two muscle groups, persistently requiring assistance in daily activities), moderate improvement (>2 grades in multiple muscle groups, occasionally requiring assistance in daily activities), marked improvement (with only mild weakness and minimal functional impairment), and complete recovery (with no symptoms or signs of muscle weakness) [23]. Treatment response on extra‐muscular activities was graded as mild improvement (by ≥20% compared to the severest condition), moderate improvement (by ≥40% compared to the severest condition), marked improvement (by ≥60% compared to the severest condition), and complete recovery (with no signs or symptoms of extra‐muscular activities) as well [21, 22]. A sufficient glucocorticoid dose was defined as at least 1 mg/kg daily at the start, tapering slowly over more than 6 months [24]. A favorable outcome was defined as marked improvement or complete recovery concerning both the muscular and extra‐muscular domains. Relapse was defined according to disease flare criteria in myositis [21].

This study was performed in accordance with the Declaration of Helsinki and approved by the Ethics Committee of Qilu Hospital (Qingdao), Shandong University, China (KYLL‐KS‐2022054). Written informed consent for tests and publication was obtained from all the participants in the present study.

### 2.2 Antibodies identification

Serum samples were collected from all 570 IIM patients and then tested by dot immunoassay (Autoimmune Myositis Profile Antibody IgG Detection Kit, MyBiotech Co., Ltd., Xi'an, China, MT559) following the manufacturer's instructions for MSAs, including anti‐signal recognition particle (SRP), anti‐3‐hydroxy‐3‐methylglutaryl‐coenzyme A reductase (HMGCR), anti‐Mi2, anti‐MDA5, antit‐TIF1γ, anti‐NXP2, anti‐SAE antibodies, eight anti‐ARS (anti‐Jo‐1, anti‐Ha, anti‐EJ, anti‐OJ, anti‐PL‐12, anti‐PL‐7, anti‐KS, and anti‐Zo) antibodies, and four MAAs including anti‐PM/Scl 75, anti‐PM/Scl 100, anti‐cN1A, and anti‐Ro52 antibodies. In this study, the anti‐OJ antibodies referred to those identified by the OJ multi‐synthetase complex, which consists of eight ARSs protein fragments: arginyl‐tRNA synthetase (RARS), glutaminyl‐tRNA synthetse (QARS), leucyl‐tRNA synthetase (LARS), Lysyl‐tRNA synthetase (KARS), Glutamyl‐prolyl‐aminoacyl‐tRNA synthetase (EPRS), aspartyl‐tRNA synthetase (DARS), methionyl‐tRNA synthetase (MARS), isoleucyl‐tRNA synthetase (IARS), and three ARS complex‐interacting multifunctional proteins (AIMP1, AIMP2, and AMIP3) components [12].

To reduce non‐specific binding, all anti‐Ha positive sera on immunoblot were further confirmed by a blocking test as in our previous study [25]. Furthermore, IP‐WB with the cultured cell extracts containing recombinant YARS protein was also performed to confirm the positivity of the anti‐Ha antibody. Detailed experimental procedures of the immunoblot, neutralizing assay, and IP can be found in Supporting Information S2.

### 2.3 Histopathological examinations

Open‐muscle biopsies were performed for routine diagnosis purposes in all IIM patients. Serial frozen sections were stained with hematoxylin and eosin (HE), anti‐CD3 mouse monoclonal antibody (clone LN10; Zhongshan Golden Bridge Biotechnology), anti‐major histocompatibility complex class‐I (MHC‐I) rabbit monoclonal antibody (clone EP1395Y; Abcam), anti‐major histocompatibility complex class‐II (MHC‐II) mouse monoclonal antibody (clone CR3/43; Dako), anti‐membrane attack complex (MAC) mouse monoclonal antibody (clone aE11; Dako), and anti‐myxovirus resistance protein A (MxA) rabbit polyclonal antibody (ab95926; Abcam).

Quantitative evaluations were performed manually in three representative fields at 200× original magnification. The scoring system used for juvenile DM with modifications was applied in the evaluation of necrotic fibers, CD3^+^ lymphocytes, and perifascicular atrophy (PFA) to reflect the severity [9, 26]. Myofiber expression of MHC‐I and MHC‐II was defined as sarcolemmal staining combined or not with sarcoplasmic staining, while MxA expression was defined as sarcoplasmic staining on non‐necrotic and non‐regenerating fibers, respectively. Perifascicular change, including PFA, PFN, MHC‐I/II, or MxA expression with a perifascicular distribution, was documented when the above pathological phenomenon was confirmed along any edge of a muscle fascicle.

### 2.4 Statistical analysis

Statistical analysis was performed using SPSS version 26.0 (SPSS, Inc., Chicago, IL, USA). Qualitative data were expressed as frequencies (percentages), whereas quantitative variables were described as mean ± standard deviation or median and interquartile range (IQR) according to data distribution. Chi‐squared test or Fisher's exact test was used to compare qualitative data. The *t* test (normal distribution) or Mann–Whitney *U* test (non‐normal distribution) was performed to compare group means or medians. *p* Values of <0.05 were considered statistically significant.

## 3 RESULTS

### 3.1 Anti‐Ha antibody prevalence in our NMD center

Nineteen out of the 570 patients with IIMs were seropositive for anti‐Ha antibody tested by immunoblot, but two of the positive serum could not be blocked by the Ha‐antigen, so that finally 17 patients were confirmed as ani‐Ha positive. Five of these 17 (29.4%, 5/17) anti‐Ha positive patients had one of other MSAs: anti‐SRP (in three patients), anti‐HMGCR (in two patients) (Figure S1); MAAs were also detected in seven patients, in whom anti‐Ha antibody was the isolated MSA (Table 1). Additionally, 49 out of the 570 IIMs patients were seropositive for one of the other six anti‐ARS autoantibodies (anti Jo‐1 in 24 patients, anti‐EJ in 9 patients, anti‐PL‐7 in 8 patients, anti‐OJ in 4 patients, anti‐PL‐12 in 3 patients, and anti‐Zo in 1 patient); among whom, 8 patients (16.3%, 8/49) had another coexisting non‐anti‐ARS MSA (anti‐SRP in 2 patients, anti‐HMGCR in 2 patients, anti‐TIF1γ in 2 patients, anti‐NXP2 in 1 patient, and anti‐MDA5 in 1 patient). Therefore, the prevalence of anti‐Ha antibody was 3.0% (17/570) in our IIMs cohorts and 25.8% (17/66) in the entire ASS group, respectively. All of the anti‐Ha positivity of our patients on immunoblot was also reconfirmed by the IP‐WB technique (Figure S2). Given the complexity of patients with double MSAs, only the patients with a single MSA (anti‐Ha or one of the other anti‐ARS antibodies) were analyzed and compared.

**TABLE 1:** Clinical and laboratory findings of 12 patients with anti‐Ha antibody as the single myositis‐specific antibody.

Pts	Sex/age at onset (year)	Disease duration (month)	RP	Arthralgia	Skin lesion			ILD	Muscle symptoms					CK (U/L)	Coexisting MAAs
					Heliotrope sigh/papule	Gottron's rash	Others		PW/DW/NW	Weakest strength	Symmetry weakness	Myalgia	Bulbar symptom		
1	F/58	24	−	−	−	−	+	−	+/−/+	5	+	−	−	1668	−
2	F/22	12	+	+	+	−	+	−	+/−/−	5	+	+	+	1365	−
3	F/4	2.5	+	−	+	+	+	+	+/−/−	5	+	−	−	113	Ro‐52
4	M/32	5	+	−	−	−	−	−	+/+/−	8	−	−	+	2642	−
5	F/48	1	−	−	−	−	−	+	+/−/−	7	+	−	+	2701	PM/Scl‐75, PM/Scl‐100
6	F/8	6	−	−	+	+	+	−	+/−/−	7	+	−	−	231	cN1A
7^a^	F/16	36	−	+	−	−	+	−	−/−/−	10	−	+	−	38	Ro‐52
8	F/33	12	−	−	−	−	+	+	+/−/+	8	+	+	−	354	cN1A
9	M/58	24	+	−	+	+	+	+	+/−/−	7	+	+	−	66	−
10	F/19	3	−	−	−	−	−	−	+/+/+	8	−	−	−	62	Ro‐52
11	M/75	1	−	−	+	−	+	−	+/−/+	8	+	+	+	722	−
12	M/57	36	−	−	−	+	+	−	+/−/+	8	+	−	−	2000	Ro‐52

Abbreviations: Abs, antibodies; CK, creatine kinase; DW, distal weakness; ILD, interstitial lung disease; MAAs, myositis‐associated antibodies; NW, neck weakness; Pts, patients; PW, proximal weakness; RP, Raynaud phenomenon; “+,” positive/present; “−,” negative/absent.

^a^ The predominant manifestation of this patient was myalgia and induration in her right calf, consistent with fasciitis in the muscle pathology.

### 3.2 Clinical features

The clinical features of 12 patients with anti‐Ha antibody as the single MSA were summarized in Table 1. There was a female predominance among anti‐Ha participants (8/12, 66.7%), including three juvenile patients (P3, P6, and P7). The median age of onset was 32.5 years, with a range from 4 to 75 years. The median time from the onset to the final diagnosis was 9 months, with a range from 1 to 36 months. Creatine kinase (CK) levels were normal or highly elevated, ranging from 38 to 2701 U/L, which were presumably associated with variable necrotic pathology in the muscle biopsies. The muscle involvement was observed in all patients both clinically and histopathologically. The most prevalent muscle weakness pattern was symmetrical‐proximal limb weakness (75%, 9/12), neck (41.7%, 5/12), and bulbar muscle involvement (33.3%, 4/12) were less common. Skin lesions were seen in nine patients (75%, 9/12), among whom DM‐specific skin rashes referring to the Heliotrope and Gottron's signs were observed in six patients (50%, 6/12). The other characteristic features of ASS were uncommon, with RP in four patients (33.3%, 4/12) and arthralgia in only two patients (16.7%, 2/12), and none of our patients suffered from fever or mechanic's hands. ILD was also infrequent in the present cohort, confirmed in only four patients (33.3%, 4/12) on high‐resolution CT, none of whom presented with prominent respiratory symptoms. None of the anti‐Ha patients had a history of concurrent malignancy.

### 3.3 Histopathological findings

**FIGURE 1:**
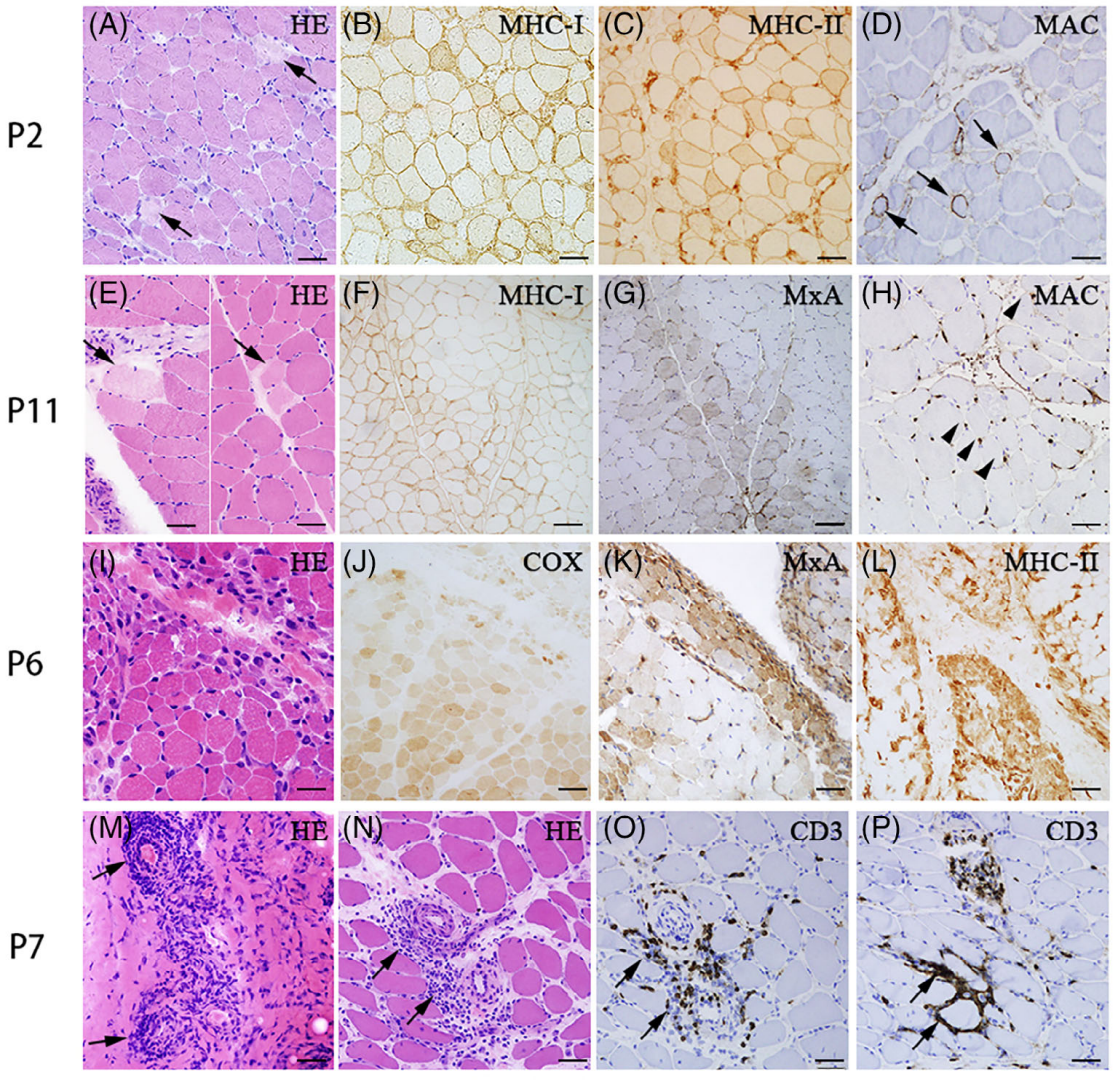
Muscle pathology. (A–D) Scattered necrotic and regenerating fibers (A, arrows, hematoxylin and eosin [HE] staining) with diffuse major histocompatibility complex class‐I (MHC‐I) (B) and major histocompatibility complex class‐II (MHC‐II) expression (C), and sarcolemmal membrane attack complex (MAC) deposition on non‐necrotic fibers (D, arrows) in P2. (E–H) Isolated perifascicular necrosis (PFN) (E, arrows, HE staining), with focal MHC‐I expression slightly intensified in the perifascicular region (F), perifascicular myxovirus resistance protein (MxA) expression (G), and MAC deposition on the capillaries (H) in P11. (I–L) Typical perifascicular atrophy (PFA) fibers (I, HE staining) with decreased cytochrome oxidase (COX) activity in the perifascicular region (J, COX staining), perifascicular MxA (K), and MHC‐II expression (L) in P6. (M–P) Perivascular inflammation in the fascia and perimysium (M and N, arrows, HE staining), focal T lymphocytic infiltrations in the perivascular spaces and endomysium, respectively (O and P, arrows, CD3 staining) in Patient 7. Scale bars: (A–E, H, I, M–P), 50 μm; (E–G, J–L) 100 μm.

The histopathological features of muscle biopsies from the above 12 anti‐Ha patients were displayed in Table 2 and Figure 1. Typical myofiber necrosis accompanied by many regenerating fibers was the most common feature observed in 58.3% (7/12) of the anti‐Ha positive patients (Figure 1A), while PFN could only be observed in one patient of them (P11, Figure 1E). Similar to other ASS subgroups, PFA was also uncommon in anti‐Ha individuals, observed in only three patients (25%, 3/12), which could be accompanied by perifascicular deficiency of COX activity (Figure 1I,J). MHC‐I expression was observed in all 12 patients, diffusely or focally, with perifascicular enhancement in 3 of them (Figure 1B,F). In comparison, MHC‐II was only upregulated in four muscles of the patients (33.3%, 4/12), diffusely or focally, with perifascicular enhancement in two of them (Figure 1C,L). Obvious MAC deposition on the sarcolemma of non‐necrotic myofibers was observed in four patients (33.3%, 4/12) (Figure 1D), three of whom obtained the highest score of 2 in the necrotic fiber domain. Capillary MAC deposition was found in only three patients (25%, 3/12) (Figure 1H). MxA expression was also observed in three muscles of the patients (25%, 3/12), with two specimens exhibiting a perifascicular pattern (Figure 1G,K). Among all our patients, muscle biopsies demonstrated variable lymphocytic infiltrates that were scattered or focally clustered in the endomysial (100%, 12/12), perimysial (83.3%, 10/12), or perivascular (75%, 9/12) domains (Figure 1M–P). Additionally, in P7, the histopathological finding of obvious perivascular inflammation in the fascia confirmed the clinical diagnosis of fasciitis (Figure 1M).

**TABLE 2:** Muscle pathological features of 12 patients with anti‐Ha antibody as the single myositis‐specific antibody.

Pts	Lymphocytic infiltrates			Decreased COX activity in PF	Punched‐out fibers	PFA score	Necrotic fiber			Immunopathological features				
	Endomysial score	Perimysial score	Perivascular				Total score	Non‐PF necrosis	PFN	MxA	MHC‐I	MHC‐II	MAC‐Sarcolemmal deposition	MAC‐capillary deposition
1	1	0	−	−	−	0	1	+	−	−	Diffuse	−	−	−
2	2	1	+	−	−	0	2	+	−	−	Diffuse	Diffuse	+	−
3	1	0	−	−	−	0	0	−	−	Focal	Diffuse	−	−	−
4	2	1	+	−	−	0	2	+	−	−	Diffuse	−	+	−
5	1	2	+	−	−	1	1	+	−	−	Diffuse	−	−	+
6	2	2	+	+	+	2	0	−	−	PF	Diffuse + PF	PF	−	−
7	2	2	+	−	−	0	0	−	−	−	Focal	−	−	−
8	2	2	+	−	−	0	2	+	−	−	Focal	Focal	−	−
9	1	1	−	−	−	0	0	−	−	−	Diffuse	−	−	−
10	1	1	+	−	−	1	0	−	−	−	Focal + PF	Focal + PF	−	−
11	1	1	+	−	+	0	1	−	+	Focal + PF	Focal + PF	−	+	+
12	2	1	+	−	+	0	2	+	−	−	Focal	−	+	+

*Note*: Scores of inflammatory domain (in 20× high power field): 0 = none or <4 cells, 1 = 4–20 cells or 1 cluster of ≥10 cells, 2 = >4 cells or ≥2 clusters in whole biopsy. Scores of PFA: 0 = absence, 1 = affecting one to two fascicle(s), 2 = affecting ≥2 fascicles. Scores of necrotic fibers (in 20 × high power field): 0 = absence, 1 = sporadic one to three fibers, 2 = scattered ≥4 fibers or clustered.

Abbreviations: COX, cytochrome oxidase; MAC, membrane attack complex; MHC‐I, major histocompatibility complex class‐I; MHC‐II, major histocompatibility complex class‐II; MxA, myxovirus resistance protein; PF, perifascicular area; PFA, perifascicular atrophy; PFN, perifascicular necrosis; Pts, patients; “+” = positive/present; “−” = negative/absent.

### 3.4 Treatment and outcomes

The detailed therapy and treatment outcomes of these 12 anti‐Ha positive patients were summarized in Table 3. The median follow‐up time was 31.5 months, with a range from 6 to 174 months. All these patients were given a sufficient dose of prednisone at 1 mg/kg, except for two juvenile patients (P3 and P6, 2 mg/kg), with or without an immunosuppressant as combination therapy. Half of them (50%, 6/12) experienced relapse during the drug discontinuation (66.7%, 4/6) or dose tapering (33.3%, 2/6) after a treatment course lasting more than 6 months; five of them experienced worsening in muscle strength, while the other (P9) reported recurrent skin lesion activity. Although three patients still suffered from different degrees of muscle weakness and functional impairment, 8 out of these 12 patients (66.7%) had achieved a favorable outcome at the last follow‐up. Additionally, one patient (P9) has achieved complete recovery in his muscle function, while the skin rashes seemed refractory in spite of being treated with a triple strategy.

**TABLE 3:** Treatment and outcomes of 12 patients with anti‐Ha antibody as the single myositis‐specific antibody.

Pts	Treatment		Follow‐up time (month)	MMT‐8		HAQ‐DI		Relapse			CK at last follow‐up	Outcomes at last follow‐up
	Therapy at beginning	Therapy at last follow‐up/duration (month)		First	Last	First	Last	Y/N	Treatment course before relapse (month)	Treatment at relapse/duration (month)		
1	Pred	None/140	174	66	78	1.25	0	Y	16	None/3	NL	Marked improvement
2	Pred, MTX	None/24	143	68	80	0.625	0	Y	24	None/6	NL	Complete recovery
3	Pred, MTX	None/48	113	62	80	1.75	0	N	‐	‐	NL	Complete recovery
4	Pred	Pred 5 mg qd/6	84	71	76	0.625	0.125	Y	11	None/2	NL	Moderate improvement
5	Pred, MTX	Pred 5 mg qod/13	69	64	76	2.25	0.25	N	‐	‐	369	Moderate improvement
6	Pred, MTX	None/6	36	64	80	2.25	0	Y	12	Pred 5 mg qd/2	NL	Complete recovery
7	Pred	Pred 15 mg qd/2.5	27	80	80	0.625	0	Y	7.5	None/1.5	NL	Complete recovery
8	Pred, TAC	Pred 5 mg qd/3	16	70	78	1.25	0	N	‐	‐	NL	Marked improvement
9	Pred, MTX	Pred 10 mg qd, MTX 7.5 mg qw, tofacitinib 0.5 g bid/6	11	72	80	1.0	0	Y	6.5	Pred 15 mg qd, MTX 7.5 mg qw/1	NL	Complete recovery in muscle function; mild improvement in skin rashes
10	Pred	Pred 10 mg qd/2	7	69	78	0.125	0	N	‐	‐	NL	Marked improvement
11	Pred, TAC	Pred 30 mg qd/1, TAC 3 mg qd/5	6.5	73	78	0.875	0.25	N	‐	‐	NL	Marked improvement
12	Pred	Pred 35 mg qd/0.5	6	73	76	1.125	0.875	N	‐	‐	NL	Moderate improvement

Abbreviations: CK, creatine kinase; HAQ‐DI, Health Assessment Questionnaire Disability Index; MMT, manual muscle testing; MTX, methotrexate; N, no; NL, normal; Pred, prednisone; Pts, patients; TAC, tacrolimus; Y, yes.

### 3.5 Comparison between anti‐Ha positive patients and other anti‐ARS patients

The median age of onset of this entire ASS cohort was 52 years, with a slight female predominance (56.6%, 30/53). Age of onset was younger in anti‐Ha patients compared to anti‐Jo‐1 subgroup and anti‐non‐Jo‐1/non‐Ha subgroup (32 vs. 51, *p* = 0.087; 32 vs. 55, *p* = 0.030), and juvenile patients could only be identified in the anti‐Ha cohort (Table 4). Of the total 53 ASS patients, muscle weakness was the most common manifestation (84.9%, 45/53) compared to the other ASS rheumatologic features including ILD (62.3%, 33/53), arthralgia (34%, 18/53) and RP (18.9%, 10/53). Bulbar symptom and neck weakness were more common in anti‐Ha patients compared to the anti‐Jo‐1 (33.3% vs. 4.5%, *p* = 0.042) subgroup and anti‐non‐Jo‐1/non‐Ha subgroup (41.7% vs. 0, *p* = 0.005), respectively. The incidence of ILD was considerably lower in anti‐Ha patients compared to the anti‐non‐Jo/non‐Ha subgroups (33.3% vs. 73.7%, *p* = 0.027). The CK levels were significantly lower in anti‐Ha individuals compared to the anti‐Jo‐1 subgroup (538 vs. 2717, *p* = 0.009). Histopathologically, PFN (identified only in one patient) was rare in anti‐Ha individuals, but not statistically different from that in anti‐Jo‐1 subgroup (8.3% vs. 45.5%, *p* = 0.068). The frequency of MHC‐II expression with or without a perifascicular pattern was significantly lower in anti‐Ha patients than that in anti‐Jo‐1 patients (33.3% vs. 81.8%, *p* = 0.014; 16.7% vs. 63.6%, *p* = 0.009). There were no other significant differences observed between the anti‐Ha and the other two anti‐ARS subgroups.

**TABLE 4:** Comparison between patients with anti‐Ha and other anti‐aminoacyl tRNA synthetase (ARS) antibodies in our center.

	All^g^ (*n* = 53)	Anti‐Ha (*n* = 12)	Anti‐Jo‐1 (*n* = 22)	Anti‐non‐Jo‐1/non‐Ha ARS (*n* = 19)^h^	Anti‐Ha versus anti‐Jo‐1	Anti‐Ha versus anti‐non‐Jo‐1/non‐Ha ARS
Clinical data						
Age at onset (year)	52 (38,59)	32 (17,57)	51 (38,62)	55 (49,67)	0.087	**0.030** *
Juvenile onset (%)	3 (5.7)	3 (25.0)	0	0	**0.037** *	**0.049** *
Female (%)	30 (56.6)	8 (66.7)	12 (54.4)	10 (52.6)	0.748	0.440
Disease duration (month)	5 (2,24)	9 (3,24)	3 (2,9)	5 (1,27)	0.211	0.610
DM skin lesion (%)^a^	16 (30.2)	6 (50.0)	6 (27.3)	4 (21.1)	0.342	0.199
Muscle weakness (%)	45 (84.9)	11 (91.7)	19 (86.4)	15 (78.9)	1.000	0.624
Bulbar symptom(%)	11 (20.8)	4 (33.3)	1 (4.5)	6 (31.6)	**0.042** *	1.000
Neck weakness(%)	12 (22.6)	5 (41.7)	7 (31.8)	0	0.842	**0.005** *
Malignancy (%)	2 (3.8)	0	2 (9.1)	0	0.529	/
Arthralgia (%)	18 (34.0)	2 (16.7)	9 (40.9)	7 (36.8)	0.289	0.424
RP (%)	10 (18.9)	4 (33.3)	1 (4.5)	5 (26.3)	**0.042** *	0.990
ILD (%)	33 (62.3)	4 (33.3)	15 (68.2)	14 (73.7)	0.051	**0.027** *
CK level (U/L)	1807 (382,4337)	538 (78,1917)	2717 (971,9002)	1787 (117,4179)	**0.009** *	0.144
Pathological features						
Necrotic fiber score	1 (0,2)	1 (0,2)	1 (0,2)	1 (0,2)	0.398	0.897
PFN (%)	14 (26.4)	1 (8.3)	10 (45.5)	3 (15.8)	0.068	1.000
Non‐PF necrosis (%)	26 (49.1)	6 (50)	9 (40.9)	11 (57.9)	0.610	0.667
PFA (%)	12 (22.6)	3 (25.0)	8 (36.4)	1 (5.3)	0.769	0.272
Endomysial inflammation score	1 (1,2)	2 (1,2)	2 (1,2)	1 (1,2)	0.718	0.187
Perimysial inflammation score	1 (1,1)	1 (1,2)	1 (1,2)	1 (1,1)	0.906	0.264
Perivascular inflammation (%)	41 (77.4)	9 (75.0)	15 (68.2)	17 (89.5)	0.982	0.350
MHC‐I (%)	50 (94.3)	12 (100)	21 (95.5)	17 (89.5)	1.000	0.510
MHC‐I, PF pattern(%)	18 (34.0)	3 (25.0)	11 (50.0)	4 (21.1)	0.293	1.000
MHC‐II (%)^b^	33 (62.3)	4 (33.3)	18 (81.8)	11 (61.1)	**0.014** *	0.136
MHC‐II, PF pattern (%)	23 (43.4)	2 (16.7)	14 (63.6)	7 (38.9)	**0.009** *	0.371
MAC, sarcolemmal (%)^c^	24 (45.3)	4 (33.3)	13 (59.1)	7 (38.9)	0.151	1.000
MAC, capillary (%)	13 (24.5)	3 (25.0)	6 (27.3)	4 (22.2)	1.000	1.000
MxA (%)^d^	6 (11.3)	3 (25.0)	2 (10.5)	1 (6.7)	0.350	0294
MxA, PF pattern (%)	5 (9.4)	2 (16.7)	2 (10.5)	1 (6.7)	0.630	0.569
Treatment and outcomes						
Prednisone monotherapy (%)^e^	21 (39.6)	5 (41.7)	10 (50.0)	6 (42.9)	0.647	0.951
Relapse (%)^f^	17 (32.1)	6 (50.0)	6 (28.6)	5 (33.3)	0.393	0.630

Abbreviations: CK, creatine kinase; DM, dermatomyositis; ILD, interstitial lung disease; MAC, membrane attack complex; MHC‐I, major histocompatibility complex class‐I; MHC‐II, major histocompatibility complex class‐II; MSA, myositis‐specific antibody; MxA, myxovirus resistance protein; PF, perifascicular area; PFA, perifascicular atrophy; PFN, perifascicular necrosis; RP, Raynaud's phenomenon.

^a^ DM skin lesions include Gottron's sign/papule and/or Heliotrope rash.

^b^ *N* = 52.

^c^ *N* = 52.

^d^ *N* = 46.

^e^ *N* = 46.

^f^ *N* = 48.

^g^ Those patients with anti‐Ha or other ARS antibodies as the single MSA.

^h^ Including anti‐EJ = 9, anti‐PL‐7 = 5, anti‐OJ = 3, anti‐PL‐12 = 1, anti‐Zo = 1.

* Bold value indicates the significant results (*p* < 0.05).

## 4 DISCUSSION

The present study is to report the clinico‐sero‐pathological profiles and long‐term follow‐up data of anti‐Ha positive patients from a large NMD center in Eastern China. The major characteristics of these anti‐Ha positive patients include a wide age range, prevalent muscle damage with uncommon PFN, scarce extra‐muscular manifestations except for skin rashes, and a good response to treatment, although relapse often occurs.

To date, there is still no standard procedure to detect anti‐Ha antibody like other anti‐ARS antibodies [27]. Immunoblot has been widely accepted as a common screening test for other anti‐ARS antibodies [27, 28]. A blocking test using a high concentration of the overexpressed cells lysate as a neutralizing solution has been applied to eliminate false positivity in some autoantibodies associated with autoimmune encephalitis [25, 29]. IP is still recognized as the gold standard of the antibody test, especially for the rare or novel ones [3]. In this study, the substantial concordance in the results of anti‐Ha antibodies between the blocking test and the IP outcomes indicated that the former might be a robust antibody validation method with a high level of reliability and practicality. The prevalence of anti‐Ha antibody was 25.8% (17/66) among the ASS patients in our NMD center, which was second only to that of anti‐Jo‐1 antibody (36.4%, 24/66). This seropositivity rate is definitely higher than that reported in prior studies, which might be attributed to the genetic predispositions (such as different HLA types) as well as variations in technical conditions and procedural specifics [13, 17, 30, 31]. Anti‐Ha antibody was mutually exclusive to other anti‐ARS antibodies in our present study, which is consistent with previous observation [30]. Moreover, the coexistence of two MSAs in IIM patients was previously reported to be 0.2%–5.3% [30, 32], while this phenomenon appeared to be more prevalent in our anti‐ARS patients (19.7%, 13/66), especially in anti‐Ha patients (29.4%, 5/17) in our NMD center. To avoid any confusion regarding those patients with double MSAs, only the patients with a single anti‐ARS antibody were included for further analysis and comparisons.

The median age at onset in our present anti‐Ha cohort was 32.5 years, which was younger than the previously reported 51.7 years in other ASS patients and 52 years in the total IIM patients [30, 33]. Additionally, three juvenile patients were identified with anti‐Ha antibody as the unique MSA in this cohort. Therefore, juvenile individuals accounted for 25% of the total 12 anti‐Ha patients, which was substantially higher compared to other ASS cohorts [8, 14, 16, 34], indicating that anti‐Ha autoantibody should be routinely detected in juvenile myositis, especially in those without other MSAs identified. Regarding the muscle symptoms, similar to the other myositis groups [35], anti‐Ha individuals consistently exhibited proximal limb weakness, mostly in a symmetrical pattern (75%), along with neck (41.7%) or bulbar muscle involvement (33.3%). Additionally, fasciitis was previously observed in approximately 33% of patients with ASS based on magnetic resonance imaging (MRI) assessment in Japan [33]. Along with our pathological findings in the present case of P7, fasciitis might be the predominant clinical presentation in anti‐Ha individuals.

Skin lesions were the most prevalent extra‐muscular manifestations in this anti‐Ha cohort (75%), among whom DM‐specific rashes were commonly seen in them (50%), which was more prevalent compared to the previous reports [27, 36]. The other extra‐muscular manifestations, such as arthralgia, RP, and fever, were uncommon compared to the previous experiences in other ASS [27, 36]. ILD has been shown to be the leading cause of mortality, with a prevalence of 67%–100% in patients with ASS [31, 37]. However, it was confirmed in only four of our anti‐Ha patients (33.3%), which was much fewer compared to those patients with anti‐Jo‐1 (68.2%) or anti‐non‐Jo‐1/non‐Ha ARS (73.7%) in our NMD center. Therefore, ILD might not be a common characteristic of the individuals with anti‐Ha antibodies. The presence of mechanic's hands was considered the cornerstone of ASS with high diagnostic specificity, especially in those with amyopathic ASS [8, 27, 33], but this characteristic sign was absent in our present anti‐Ha cohort. The unusual extra‐muscular symptoms except for skin lesions in anti‐Ha patients reconfirmed the clinical heterogeneity among patients with different anti‐ARS autoantibodies [27].

In regard to the muscle biopsies, PFN and perifascicular MHC‐II expression were considered highly specific pathological features for anti‐Jo‐1 ASS and could be also observed in other anti‐ARS patients, such as anti‐Jo‐1, anti‐PL‐7, and anti‐OJ patients [7, 8, 16]. However, PFN was seen in only one of the present anti‐Ha cohort. This discrepancy might be because of the fact that PFN is preferentially present in some specific anti‐ARS subgroup [7, 8, 16]. Although there were heterogeneous histological manifestations, a necrotizing myopathy without PFN seemed to be the most common pathological manifestation (50%, 6/12) in our present anti‐Ha cohort. This was actually also consistent with a previous study focusing on the other anti‐ARS myopathology (46.2%) [16]. Myofiber MHC‐II expression, especially with a perifascicular pattern that is attributable to interferon γ in the microenvironment of myofibers, is recognized as a diagnostic biomarker for ASS myopathy with high specificity (95.4%) and sensitivity (61.2%) [10, 16]. Unexpectedly, the frequency of myofiber MHC‐II expression in our anti‐Ha patients (33.3%) was much lower than that in other anti‐ARS individuals (60.4%) [16]. Therefore, MHC‐II expression seemed not to be available to characterize anti‐Ha myositis. This difference in MHC‐II expression between anti‐Ha and other ASS myopathies might indicate a specific autoimmune mechanism in anti‐Ha myositis, possibly involving different cytokine profiles. Sarcoplasmic MxA expression, which has been suggested to be a specific marker of DM [9, 16], was detected in three (25%, 3/12) of the present anti‐Ha patients. Actually, MxA positivity was not rare among the overall ASS patients in our NMD center (11.3%), substantially higher than that of the other study (1.4%) [16, 18]. These findings may be attributed to the varying sensitivities and specificities of different antibody clones, as well as the moderate levels of interferon I‐inducible genes observed in ASS [16, 38]. As a result, our study suggested that a necrotizing myopathy with less frequent PFN and MHC‐II expression might be the most common pathological prototype of anti‐Ha myositis.

In our present study, most of the anti‐Ha patients had a good prognosis, irrespective of their histopathological manifestations. A previous study indicated that muscle weakness at the diagnosis of ASS was associated with a better prognosis, probably because of the timely and aggressive treatment [28]. Although we could not exclude the possibility of patient selection bias in our NMD center, the primary muscle impairment and milder lung involvement might be one explanation for the favorable outcomes of our anti‐Ha patients. It is noteworthy that the relapse rate of muscle weakness was also reported in 50% (6/12) of this anti‐Ha cohort during drug tapering or discontinuation, which is even higher than that of ILD in ASS (36.3%) [39]. Although the high rate of relapse, most of them (75%, 9/12) finally achieved a favorable outcome in their muscle function at last follow‐up. Skin lesions might be refractory in some anti‐Ha patients like P9 in our cohort. These results suggested the need for a more cautious tapering strategy or an early combination therapy to control the possible long‐term disease activity in these anti‐Ha patients [40].

The limitations of this study include the following aspects: First, this is a retrospective analysis; clinical information was recollected through the medical recording and the patients' recalling, so missing data and statistical bias would be inevitable. Second, the follow‐up in these patients was irregular so that the exact drug sustenance and their relationship with relapse could not be analyzed. At last, this is a single NMD center cohort with a limited number of patients; a larger multicenter and multidisciplinary study is warranted to confirm and extend our findings.

In conclusion, anti‐Ha antibody is not as rare as previously believed and could coexist with other MSAs. The anti‐Ha patients generally display muscle weakness with a typical limb‐girdle pattern, while extra‐muscular manifestations except for the cutaneous lesions are unusual. The most common pathological picture for anti‐Ha patients is a necrotizing myopathy with less frequent PFN and MHC‐II expression.

## AUTHOR CONTRIBUTIONS

Bing Zhao: Writing–Original draft preparation. Ying Hou: Data curation. Kai Shao, XiaoTian Ma: Investigation, Resources. YaPing Yan: Antibody detection guidance. Jian‐Qiang Lu, LiNing Zhang, TingJun Dai: Writing–Reviewing and Editing. Wei Li, ChuanZhu Yan: Conceptualization, Methodology, Funding acquisition.

## CONFLICT OF INTEREST STATEMENT

The authors have declared no conflicts of interest.

## Supporting information


**Figure S1.** Representative results of double MSAs positivity confirmed by blocking test on immunoblot. (A–D) Double positivity with anti‐Ha and anti‐SRP antibodies revealed on the test strip 1 (A); the anti‐Ha dot could be blocked by the high concentration of Ha‐antigen (B); the anti‐SRP dot could be blocked by the high concentration of SRP‐antigen (C); while both anti‐Ha and anti‐SRP dots couldn't be neutralized by the control protein (D). (E–G, I–K) Double positivity with anti‐Ha and anti‐HMGCR antibodies revealed on test strip 1 (E, on test strip 1) and 2 (I, on test strip 2), respectively. The anti‐Ha dot could be blocked by the Ha‐antigen (F, on test strip 1) but not the control protein (G, on test strip 1), and the anti‐HMGCR dot could be blocked by the HMGCR‐antigen (J, on test strip 2) but not the control protein (K, on test strip 2). (H, L) The visual representation of myositis specific and associated antigens on the test strips in our study. The blocking protein used as control was glutamic acid decarboxylase; and the dot in the upper left corner is a positive indicator of good quality control (arrowheads). HMGCR, 3‐hydroxy‐3‐methylglutaryl‐coenzyme A reductase; MSAs = myositis specific antibodies; SRP, signal recognition particle.
**Figure S2**. Immunoprecipitation‐western blotting results. Immunoprecipitants derived from the serum of Patient 5 and HEK293T cell extracts were detected with anti‐Ha monoclonal antibody (arrowhead).

**Data S2.** Supporting Information.

## Data Availability

Anonymized data not published within this article will be made available by any qualified investigators from the corresponding author on reasonable request.
