# Supplementary material for: Clinico‐sero‐pathological characteristics of anti‐Ha antisynthetase syndrome
Source: Brain Pathol. 2024 Nov 18;35(3):e13319. doi: 10.1111/bpa.13319 (PMC11961205; doi:10.1111/bpa.13319)
Supplement: Supplementary file 1 — Figure S1. Representative results of double MSAs positivity confirmed by blocking test on immunoblot. (A–D) Double positivity with anti‐Ha and anti‐SRP antibodies revealed on the test strip 1 (A); the anti‐Ha dot could be blocked by the high concentration of Ha‐antigen (B); the anti‐SRP dot could be blocked by the high concentration of SRP‐antigen (C); while both anti‐Ha and anti‐SRP dots couldn't be neutralized by the control protein (D). (E–G, I–K) Double positivity with anti‐Ha and anti‐HMGCR antibodies revealed on test strip 1 (E, on test strip 1) and 2 (I, on test strip 2), respectively. The anti‐Ha dot could be blocked by the Ha‐antigen (F, on test strip 1) but not the control protein (G, on test strip 1), and the anti‐HMGCR dot could be blocked by the HMGCR‐antigen (J, on test strip 2) but not the control protein (K, on test strip 2). (H, L) The visual representation of myositis specific and associated antigens on the test strips in our study. The blocking protein used as control was glutamic acid decarboxylase; and the dot in the upper left corner is a positive indicator of good quality control (arrowheads). HMGCR, 3‐hydroxy‐3‐methylglutaryl‐coenzyme A reductase; MSAs = myositis specific antibodies; SRP, signal recognition particle. Figure S2. Immunoprecipitation‐western blotting results. Immunoprecipitants derived from the serum of Patient 5 and HEK293T cell extracts were detected with anti‐Ha monoclonal antibody (arrowhead). [file BPA-35-e13319-s002.docx]

**
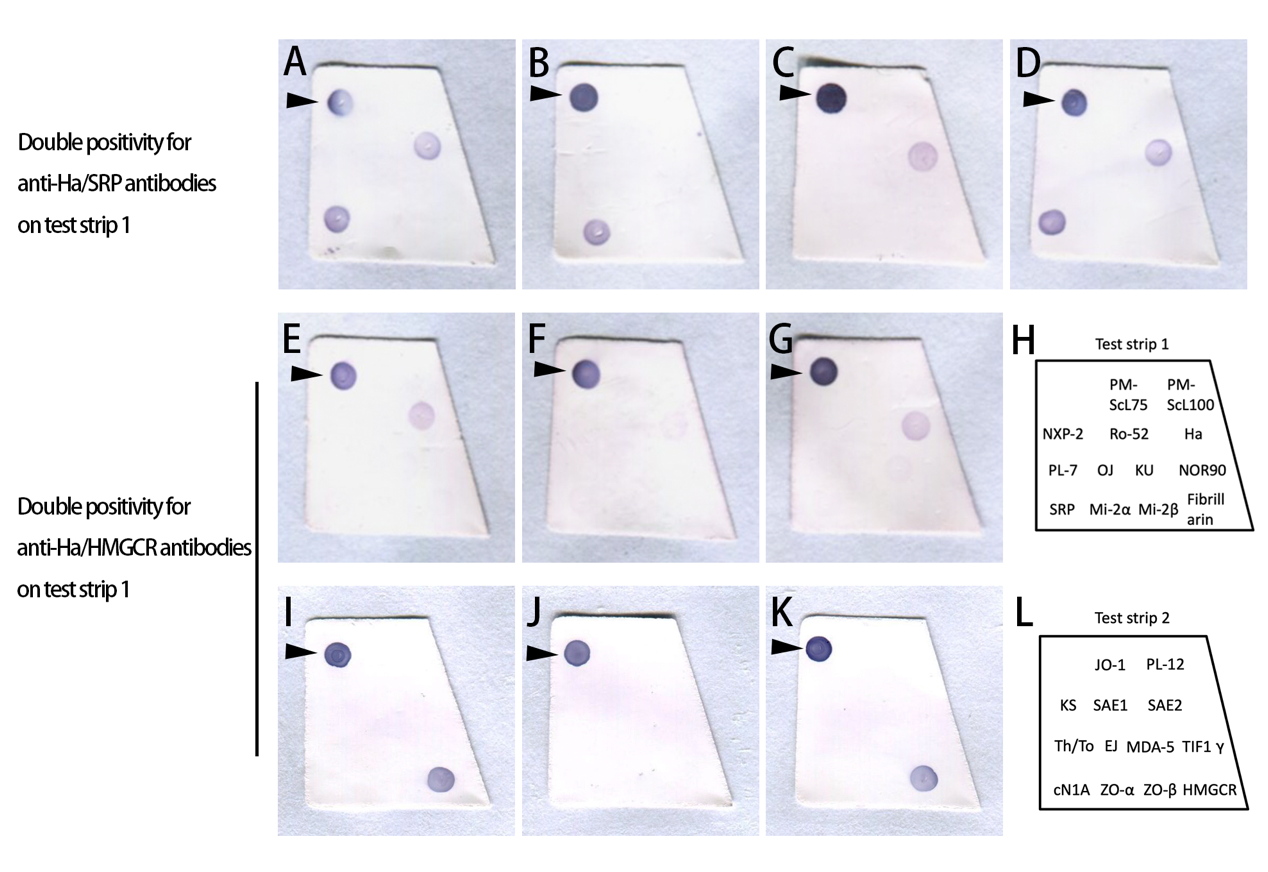
**

**Supplementary Figure S1**. **Representative results of double MSAs positivity confirmed by blocking test on immunoblot.** (A-D) Double positivity with anti-Ha and anti-SRP antibodies revealed on the test strip 1 (A); the anti-Ha dot could be blocked by the high concentration of Ha-antigen (B); the anti-SRP dot could be blocked by the high concentration of SRP-antigen (C); while both anti-Ha and anti-SRP dots couldn’t be neutralized by the control protein (D). (E-G, I-K) Double positivity with anti-Ha and anti-HMGCR antibodies revealed on test strip 1 (E, on test strip 1) and 2 (I, on test strip 2), respectively. The anti-Ha dot could be blocked by the Ha-antigen (F, on test strip 1) but not the control protein (G, on test strip 1), and the anti-HMGCR dot could be blocked by the HMGCR-antigen (J, on test strip 2) but not the control protein (K, on test strip 2). (H, L) The visual representation of myositis specific and associated antigens on the test strips in our study. The blocking protein used as control was glutamic acid decarboxylase; and the dot in the upper left corner is a positive indicator of good quality control (arrowheads). Abbreviation: MSAs=myositis specific antibodies; SRP=signal recognition particle; HMGCR=3-hydroxy-3-methylglutaryl-coenzyme A reductase.


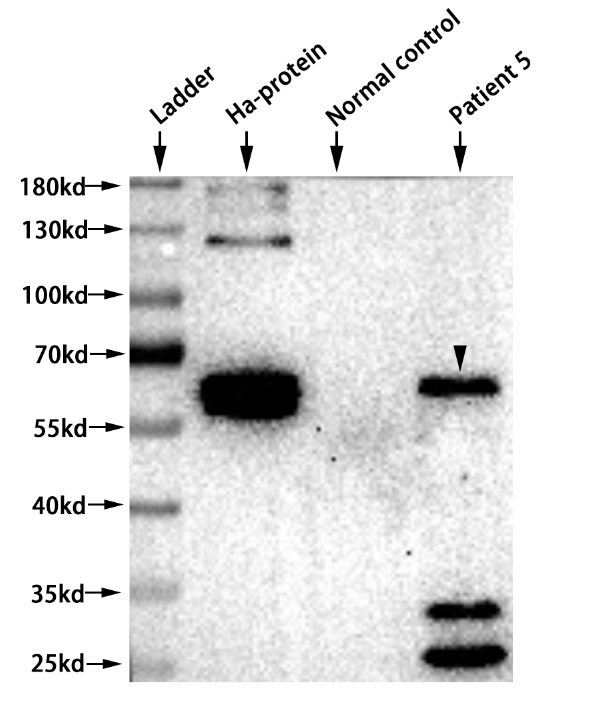


**Supplemental Figure S2. Immunoprecipitation-western blotting results.** Immunoprecipitants derived from the serum of patient 5 and HEK293T cell extracts were detected with anti-Ha monoclonal antibody (arrowhead).
