# Supplementary material for: Clinico‐sero‐pathological characteristics of anti‐Ha antisynthetase syndrome
Source: Brain Pathol. 2024 Nov 18;35(3):e13319. doi: 10.1111/bpa.13319 (PMC11961205; doi:10.1111/bpa.13319)
Supplement: Supplementary file 2 — Data S2. Supporting Information. [file BPA-35-e13319-s001.doc]

**Supplementary Data S1**

1. **Description of specific experimental steps of the immunoblot assay for MSAs detection**

**Purification of recombinant proteins** CDS sequences of myositis-specific antigens were inserted into the prokaryotic expression plasmid to construct recombinant vectors (Supplementary Table S1). The E.coli strain BL21(DE3) transformed with recombinant vectors were cultured in Luria-Bertani (LB) medium containing ampicillin (50μg/ml) or kanamycin (30μg/ml) at 37℃ with shaking at 200 rpm. When OD_600_ of culture reached 0.6, recombinant protein expression was induced by adding isopropyl-beta-D-thiogalactopyranoside (IPTG) to a final concentration of 1mM and cultivating for 4h. BL21(DE3) cell lysates preparation and protein purification procedure were performed under native conditions as specified in the manufacturer's instructions (Qiagen). Briefly, BL21 (DE3) cell containing each recombinant protein were harvested and re-suspended in lysis buffer (50mM NaH_2_PO_4_, 300mM NaCl, 10mM imidazole, pH8.0). The bacteria were lysed by sonication and centrifuged at 10,000g for 20min at 4℃. The supernatant were applied to Ni-NTA Magnetic Agarose Beads (Qiagen), washed with binding buffer（50mM NaH_2_PO_4_, 300mM NaCl, 10mM imidazole, pH8.0）and eluted with elution buffer (50mM NaH_2_PO_4_, 300mM NaCl, 500mM imidazole, pH8.0). All recombinant proteins solution were dialyzed against PBS buffer, pH 7.2 and then stored at -20℃.

**Test strip preparation** Each recombinant proteins (50ng in 1ul) solution was spotted onto a nitrocellulose membrane (GE life sciences, USA) and dried at room temperature for 30 minutes. The test strip (antigen-coated NC membrane) was sealed and stored at 4°C for long term storage.

**Immunoblot test procedure** Each sample of the patient serum (30μl) was 1:10 diluted with PBS and added to test strip in a 12-well plate. Thirty minutes later, the sample mixture was removed, and the test strip was washed two times with PBST for 5 minutes each time. The AP-goat anti-human IgG gamma secondary antibody (Jackson ImmunoResearch Laboratories, PA, USA) was 1:2000 diluted with PBS and added in the wells. After incubation for 30minutes, the strips were washed two times with 150 mM Nacl, and substrate buffer containing NBT/BCIP (AMRESCO, lnc, PA, USA) was added to the well. The color development was completed within 4 minutes. The strip was washed with ddH_2_O to terminate the reaction and dried at 40-60℃ for 10 minutes.

**Neutralizing test based on the immunoblot assay** The positive sera, which have been diluted with PBS at a ratio of 1:300, were incubated with the recombinant Ha protein (9μg for 300ul diluted serum) or an unrelated protein (glutamic acid decarboxylase, 9μg for 300ul diluted serum) as control; after incubating for 5 minutes at room temperature, a normal blot test was performed using the serum and the sera-protein mixture respectively.

1. **Description of specific experimental steps of the immunoprecipitation assay for anti-Ha antibody detection**

**Preparation of YARS (Ha) overexpression cells** Briefly, 36 hours after the transfection with plasmid (pcDNA3.1) carrying human tyrosyl-tRNA synthetase gene or empty plasmid as control, HEK293T cells were fixed with 4% paraformaldehyde and permeabilized with 0.4% Triton X-100 in phosphate buffered saline (PBS) for 10 mins. The fixed cells were stained with the commercial anti-YARS (HPA018954, sigma) antibody for 30 mins at room temperature to indicate YARS overexpression. Immunostained cells were screened with fluorescence microscope (EVOS M5000, Thermo Fisher Scientific, USA).

**Immunoprecipitation procedure**

- Preparation of the Ha-protein samples: collect the above Ha-overexpression cells. Sonicate the sample for 2 minutes at 5% power with a lysis buffer (mixture of 0.5% PBS Tween20 and protease inhibitor). Extract the Ha protein at 4℃ by centrifuging the lysed sample at 15000 rpm for 10min from the supernatant.
- Immunoprecipitation: after diluting the sera from the patients and the normal control at a ratio of 1:200 with PBS buffer, take 1ml from each sample and incubate them with 50μl of the above supernatant ( containing 10 μg/μl of Ha protein) for two hours. Then, incubate the protein A/G agarose beads with the above prepared mixture, refrigerate it at 4℃ overnight, and briefly centrifuge to collect the agarose beads.
- Washing and elution: wash the beads three times with 0.5% PBS Tween20. The collected agarose beads are boiled with 100μl of 1x loading buffer and then centrifuged to obtain the supernatant for use in the following Western Blotting (WB).

**Western Blot procedure** The immunoprecipitated protein mixtures are analyzed using a common WB procedure. The commercial anti-YARS (HPA018954, sigma) antibody is used to detect whether the Ha-antigen has been captured by the patients’ samples.

Supplementary Table S1. Recombinant vectors information

| Gene name | Insert vector | Resistances | Recombinant vector name |
| --- | --- | --- | --- |
| SRP54 | pET28 | kanamycin | pET28/SRP54 |
| HMGCR | pET28 | kanamycin | pET28/HMGCR |
| Mi-2(Mi-2α&Mi-2β) | pET28 | kanamycin | pET28/Mi-2 |
| MDA5 | pGEX-4T | ampicillin | pGEX-4T/MDA5 |
| TIF1γ | pGEX-4T | ampicillin | pGEX-4T/TIF1γ |
| NXP2 | pET28 | kanamycin | pET28/NXP2 |
| SAE(SAE1&SAE2) | pET28 | kanamycin | pET28/SAE |
| Jo-1(HARS) | pET28 | kanamycin | pET28/Jo-1 |
| EJ(GARS) | pET28 | kanamycin | pET28/EJ |
| PL-12( AARS ) | pET28 | kanamycin | pET28/PL-12 |
| PL-7(TARS) | pET28 | kanamycin | pET28/PL-7 |
| KS(NARS) | pET28 | kanamycin | pET28/KS |
| ZO(ZO-α&ZO-β) | pET28 | kanamycin | pET28/Zo |
| OJ(IARS) | pET28 | kanamycin | pET28/OJ |
| cN1A | pET28 | kanamycin | pET28/cN1A |
| Ha(YARS ) | pET28 | kanamycin | pET28/Ha |
| Ro52 | pET28 | kanamycin | pET28/Ro |
| PM-scl75 | pET28 | kanamycin | pET28/scl75 |
| PM-scl75 | pET28 | kanamycin | pET28/scl75 |
